# Boat noise impacts risk assessment in a coral reef fish but effects depend on engine type

**DOI:** 10.1038/s41598-018-22104-3

**Published:** 2018-03-01

**Authors:** Mark I. McCormick, Bridie J. M. Allan, Harry Harding, Stephen D. Simpson

**Affiliations:** 1 ARC Centre of Excellence for Coral Reef Studies, and College of Marine and Environmental Sciences, James Cook University, Townsville, Queensland 4811 Australia; 2 School of Biological Sciences & Cabot Institute, University of Bristol, 24 Tyndall Avenue, Bristol, BS8 1TQ UK; 3 Biosciences, College of Life and Environmental Sciences, University of Exeter, Geoffrey Pope, Stocker Road, Exeter, EX4 4QD UK; 4 Present Address: Institute of Marine Research, Bergen, Norway

## Abstract

Human noise pollution has increased markedly since the start of industrialization and there is international concern about how this may impact wildlife. Here we determined whether real motorboat noise affected the behavior, space use and escape response of a juvenile damselfish (*Pomacentrus wardi*) in the wild, and explored whether fish respond effectively to chemical and visual threats in the presence of two common types of motorboat noise. Noise from 30 hp 2-stroke outboard motors reduced boldness and activity of fish on habitat patches compared to ambient reef-sound controls. Fish also no longer responded to alarm odours with an antipredator response, instead increasing activity and space use, and fewer fish responded appropriately to a looming threat. In contrast, while there was a minor influence of noise from a 30 hp 4-stroke outboard on space use, there was no influence on their ability to respond to alarm odours, and no impact on their escape response. Evidence suggests that anthropogenic noise impacts the way juvenile fish assess risk, which will reduce individual fitness and survival, however, not all engine types cause major effects. This finding may give managers options by which they can reduce the impact of motorboat noise on inshore fish communities.

## Introduction

Sound is a fundamentally important sensory cue for marine organisms^1^, but in most inshore waters the natural soundscape generated by biological and physical sources is now polluted by anthropogenic noise^2,3^. As growing human populations often cluster around coasts, our use of continental shelf waters has dramatically increased worldwide over the last 70 years^4^. For instance, there were an estimated 15.8 million motorboats in the USA in 2014^5^ and 89,464 commercial ships transported 9.84 billion tons of cargo globally in 2015^6^. Given the prevalence of vessel traffic and accompanying engine noise, it is crucial that we have a clear understanding of the potential impacts of vessel noise on marine organisms, so that potential threats can be managed effectively. Many countries are legislating against marine noise pollution (e.g., US National Environment Policy Act; European Commission Marine Strategy Framework Directive), but currently the scientific information to support policy initiatives is lacking^7^.

Anthropogenic noises are often louder, more frequent and different in character compared with natural acoustic sounds^8–10^. Research suggests that marine organisms hear and produce sound at frequencies that directly overlap with those emitted by the operation of a variety of motorboats, ships, seismic surveys, and pile driving operations^2,4^. When anthropogenic noise competes with naturally-produced sound it can lead to the masking of communication and sensory confusion in organisms. Fish and invertebrates produce sounds during reproductive behaviour, territorial defence and predator avoidance^11,12^. Fishes also use this biological and physical sound for orientation^12–14^, and to inform important decisions, such as where to settle at the end of the larval phase^15–19^. Currently little is known of how the alteration of natural soundscapes by anthropogenic noise influences the information-base upon which behavioural decisions are made.

Noise that alters the ability of fishes to get information through sound, or disrupts the auditory system, may have ecological consequences for each part of a fish’s lifecycle. While these have been explored to a limited extent using noise playback in laboratory conditions, we know little of the effect of anthropogenic noise on marine organisms within a natural setting. Empirical studies that have examined the interplay of other sensory modalities (in particular sight and smell; e.g.^20,21^), suggest that a reduction in the efficacy of hearing may lead to sensory compensation, with a rebalance of information obtained from other senses, such as vision and olfaction^22^. The loss or degradation of information from hearing may lead to increased vigilance, which occurs at the expense of other fitness-associated behaviours such as foraging, territorial maintenance, courting and effective reproduction^23^.

Most marine organisms have complex life cycles, where embryos develop into dispersive larvae that then metamorphose and settle to join the juvenile and/or adult population. It is during this transitional stage between environments when anthropogenic noise can have a particularly strong influence on survivorship and cohort success^19,24,25^. Because the environmental conditions required by the settling juveniles are patchy and the predator composition is unpredictable, juveniles must rapidly learn to recognise a novel suite of reef predators. Learning occurs by either direct experience with a predator, or alternatively, indirectly through public information. One particularly important mechanism is learning through the concurrence of a damage-released olfactory cues (or chemical alarm odour) from a conspecific and a sensory cue associated with a predator (whether it is their smell, sight, vibration pattern or sound^20,26,27^). Anything that alters the ability of an organism to use sensory information to assess or judge risk will modify their behavioural decisions and probability of death^2,21,28^.

Research on the effects of anthropogenic sound on fishes suggests that the noise produced by different types of anthropogenic disturbance will influence different fish species in different ways. This is hardly surprising given species-specific sensitivities to sound^29,30^, differences in their use of sound to obtain and send information^29^, and differences in the characteristics of the noise produced by a variety of anthropogenic sources^2^. Despite these predictions, no studies exist that compare the impact of different motor types on natural fish behavior in an experimentally rigorous way. Information on how different noise sources affect fish is important as it gives resource managers options that may facilitate the reduction of noise pollution and its impact on wildlife.

The present study determined whether real motorboat noise affected the behavior, space use and escape response of a juvenile damselfish in the wild, and explored whether they could use chemical alarm odours to effectively assess risk in the presence of motorboats. Boats were powered by one of two types of motors: 2-stroke or 4-stroke 30 hp outboard engines. Four-stroke outboards tend to be more fuel efficient and quieter than 2-stroke outboards, but they also tend to cost considerably more. In 2007, it was estimated that 90% of the small motorboats in use around Australia had 2-stroke outboard engines^31^.

## Materials and Methods

### Study location and species

The Great Barrier Reef (GBR) is composed of ~2,900 coral reefs over 20,053 km^2^ stretching 2000 km along the north-eastern coast of Australia. Many of these reefs, particularly the inshore reefs, are regularly visited by the 90,000 recreational motorboats that were registered in Queensland in 2014. In 2012–13 there were 9,619 ships (>50 m) that used the inner lagoon as a waterway^32^. It is forecast that this may increase by 250% in the next 20 years^32^. The Lizard Island study location (14°41′S, 145°27′E) on the northern GBR, Australia, represents a section of the GBR lagoonal basin with low vessel traffic, with the closest town being Cooktown 90 km south.

The model species used for the study was the Ward’s damselfish, *Pomacentrus wardi* (Pomacentridae), a site-attached damselfish that is common on the shallow reefs of the Indo-Pacific (Supplementary Fig. S1d). Adults and juveniles occur in shallow lagoons, where they inhabit the reef edge or reef top associated with rubble and soft coral^33^. Larval duration is 16 to 21 d and fish are 13 to 14 mm standard length (SL) at the end of the larval stage^34^. Newly-settled fish are found as solitary individuals associated with conspecific adults and sub-adults, where they are subject to an array of resident and transient predators. Previous studies at this location have shown that similar species suffer variable but high mortality rates of up to 100%^35,36^. Juveniles used in the current study were caught overnight in light traps^37^ deployed at least 50 m off the reef edge prior to dusk and collected at dawn. At capture these fish are at the end of their larval phase and undergo their colour metamorphosis in the light traps^38^. They have yet to experience the benthic reef environment and are largely naïve to reef based predators^39^. Once captured fish were transported to the research station and placed in 30 L plastic aquaria without aeration. Flow-through seawater entered the holding tanks by means of submerged pipes to reduce noise levels within tanks. Fish were kept in the tanks for 24 h prior to their use in the field experiment.

The focal species has been shown to possess an innate antipredator response to conspecific alarm odours, involving a reduction in activity, space use, foraging and often an increase in shelter use^39^, typical of most damselfishes^40^.

The present study was conducted at Casuarina beach on the leeward side of Lizard Island (14°41′S, 145°27′E), northern Great Barrier Reef, Australia in 2–3 m of water on a shallow sloping beach made up of coarse coral sand interspersed with lagoonal habitat patches comprised of dead and live coral (20–30 m in diameter).

### Soundscapes

The soundscapes to which fish were experimentally exposed represent a series of passages of single boats past a stationary study site (Fig. S1a) over a ~9 min period (detailed below). It is unknown how this level of motorboat noise pollution compares to the distribution of noise levels to which fish within inshore waters are typically exposed, because relatively few studies have recorded background vessel noise levels. Logic predicts that noise pollution from vessels will be site-specific, and highly variable in space and time, with diel, daily and seasonal patterns (e.g.,^41^). It is likely that the noise levels used in the current study are equivalent to a shallow boat channel, or entrance to a small marina.

**Figure 1:**
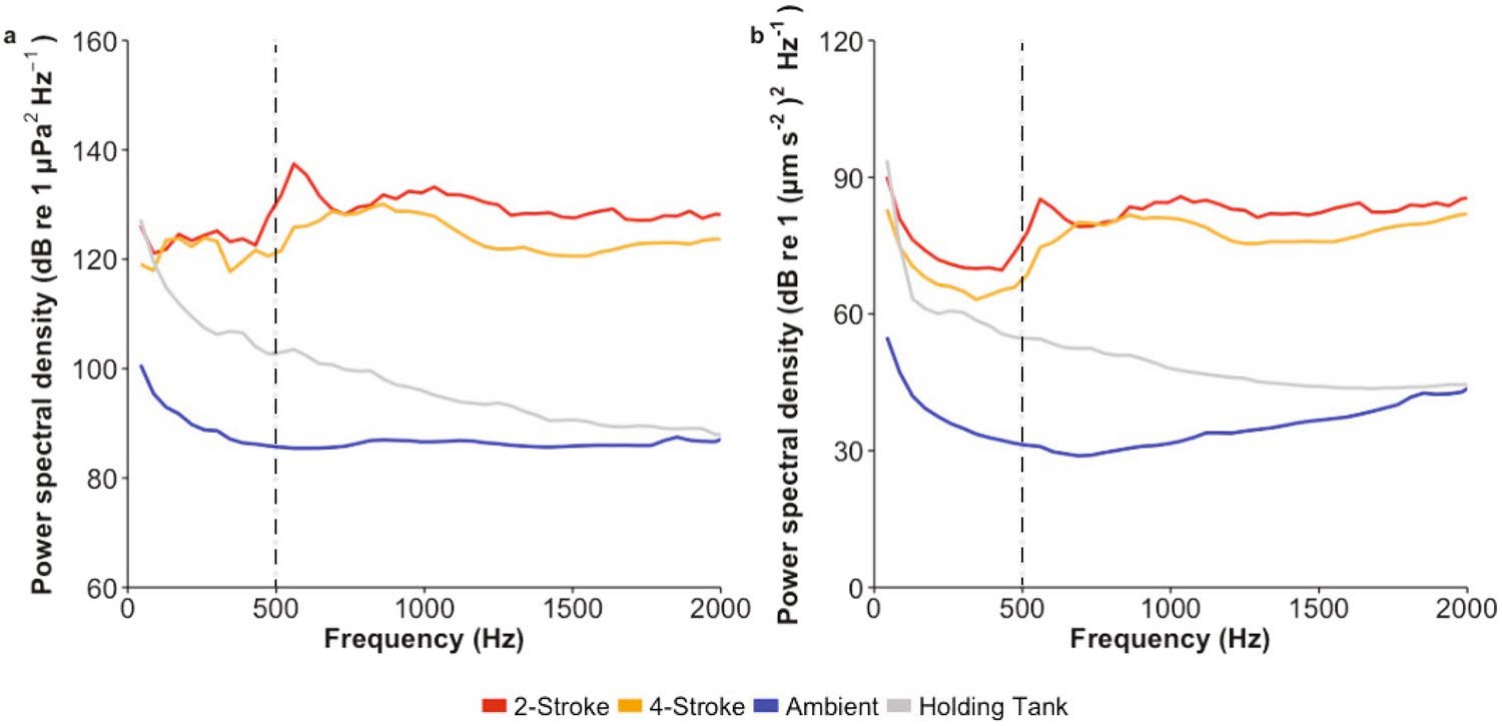
Analysis of acoustic conditions. Spectral content of ambient reef and motorboat (2-stroke and 4-stroke engine types) field recordings, as well as holding tank conditions measured in both: (**a**) acoustic pressure and (**b**) triaxial particle acceleration. Mean power spectral density of all conditions are shown. Mean spectral density levels for the two engine types were determined by combining two 7 s extracts of boat passes from each boat used for the two engine types (replicates: 2-stroke = 5, 4-stroke = 4). Ambient levels were determined by combining two 7 s extracts from each of the 8 different ambient recordings and the holding tank recording analysed using two 7 s extracts. The zone between 0–500 Hz is marked to emphasise the region of most sensitive hearing for these damselfish^70^. Sounds were analysed using the PaPAM acoustics analysis package (see^71^) using MATLAB v2014a, fft length = 1024, Hamming evaluation window, 50% window overlap, 0–3 kHz).

Sound recordings were made for all parts of the experimental process from transport to maintenance within holding tanks, through to acoustic exposure conditions with the experimental motorboats. Acoustic pressure and particle acceleration were measured within the holding fish tanks, and at the site of the behavioural studies (Fig. 1). The acoustic spectra of nine 5 m aluminium boats with either a 30 hp 2-stroke (Suzuki DT30) or 4-stroke (Suzuki DF30A) outboard engine types were characterized. The hull designs were the same for all boats. Five boats were powered by 2-stroke outboards, while four were powered by 4-stroke outboards. Boats followed the same approximate path throughout the experimental study (path shown in Fig. S1). Ambient reef sound, without boat noise was also recorded for the study site and used for comparison. Acoustic pressure and particle acceleration were measured using a calibrated triaxial accelerometer with inbuilt omnidirectional hydrophone (M20-040; sensitivity 0–3 kHz; Geospectrum Technologies, Dartmouth, Canada) and a digital 8 track field recorder (Zoom F8 field recorder, sampling rate 48 kHz (44.1 kHz holding tank recording), Zoom Corporation, Tokyo, Japan). All recording levels used were calibrated using pure sine wave signals from a function generator with known voltage recorded in line on an oscilloscope. Boat passes of a standardised length (14–16 s) from each engine type were sampled and subsequently analysed using two 7 s extracts from each boat recording appended together to determine mean spectral density levels across each engine type.

To illustrate how noise changed over the passage of the boat past the experimental site (Fig. S1a) in both the pressure and particle acceleration domains, noise levels were plotted across frequencies over a 30 s period centred on the time of maximum amplitude.

### Risk assessment experimental protocol

Experimental trials were conducted on small coral patches constructed of a combination of live *Pocillopora damicornis* and dead coral (~3:2 ratio) (18 × 18 × 15 cm) sitting in 2–3 m water (depending on tide) on a substratum of coral sand. Motorboat or ambient treatments were regularly dispersed among trials, so tide had a similar effect on both noise treatments.

Five metre aluminum boats with either a 30 hp 2-stroke engine or a 30 hp 4-stroke outboard engine (characterised above) were used as the sources of boat noise for the study. Vessels had exactly the same hull design and so differed in motor type alone (Fig. S1b). For each motor type, two sound treatments were undertaken: motorboat driving 20–200 m away from the experimental patch reefs in a figure-of-eight course (Fig. S1a), or ambient reef sound. Under each sound treatment juvenile fish were exposed to one of three odours: (a) damage-released odours from a conspecific (chemical alarm odours); (b) skin extract controls from a phylogenetically and ecologically distant heterospecific fish (*Apogon fragilis*, Apogonidae: controlling for a response to the damaged skin of any fish); (c) seawater (injection control). Fish were only used once. This gave a 2 (Acoustic treatment) × 3 (Odour) ANOVA design. Due to the logistics of the availability of the motorboats, the studies using the 2- and 4-stroke engines were undertaken separately, although studies were only temporally separated by 2 days.

To prepare the damage-released odours underwater, a small fish (a recently-settled juvenile *P*. *wardi* for the conspecific odours, or *A*. *fragilis* for the heterospecific odours) was placed into a 75 × 125 mm clip-sealed bag filled with ~200 ml of seawater. Fish were euthanized by a quick blow to the brain case and the epidermis of the fish was lacerated (~10 times) using a scalpel blade that had been placed in the bag. Donor fish were of similar size (12–14 mm standard length) to focal individuals.

Light trap caught juvenile *P*. *wardi* were individually released onto the patch reefs and given 10 min to habituate under ambient reef sound. Ten minutes has been found to be sufficient for newly metamorphosed damselfishes to explore, adopt a specific position on the patch reef, and start feeding^39^. Our previous studies have shown that the behavior and space use by the recently settled damselfish is remarkably consistent over time periods up to 5 days^34,42^. For the motorboat noise trials fish were given between 1 and 3 min of motorboat noise prior to undertaking the pre-stimulus behavioural assessment, this was followed by the injection of one of the three odours. The odours were delivered onto the patch reefs by a 2 m long plastic tube positioned up-current of the patch (Fig. S1c). Odours (60 ml) were slowly injected via a syringe, and then flushed with a further 60 ml of ambient seawater. The observer was blind to the contents of the odour in the syringe. The space use and behavior of the focal fish was assessed 3 min prior to injection of one of the three odour treatments, and 3 min afterwards. The difference between the before and after observations, compared to the variability found among the controls, gave a measure of the influence of the noise and odour treatments on the fish.

The behaviour of fish was assessed by an observer on snorkel (MIM) positioned at least 1.5 m away from the patch reef, with the aid of a magnifying glass. Three aspects of activity and behaviour were estimated for each 3 min sampling period by keeping track of where the fish travelled and knowing the dimensions of the reef: total distance moved (cm), maximum distance ventured from shelter (Max DV; cm) and boldness. Boldness was assessed using a continuous scale between 0 and 3 where: 0 is hiding in hole and seldom emerging; 1 is retreating to hole when scared and taking more than 5 sec to re-emerge, weakly or tentatively striking at food; 2 is shying to shelter of patch when scared but quickly emerging, purposeful strikes at food; and 3 is not hiding when scared, exploring around the coral patch, and striking aggressively at food^43^. This measure has been shown to be repeatable and linked to survival in other studies using newly settled damselfishes^44–46^. Three minute behavioural assessments have previously been be found to be sufficiently long to obtain a representative estimate of an individual’s behaviour at this ontogenetic stage^42,47^.

### Effect of boat noise on fish startle response

To determine whether the noise of either 2-stroke or 4-stroke engines affected the ability of juvenile *P*. *wardi* to respond to a startle stimulus, fish were placed in 80 ml plastic jars and exposed to a startle stimulus while in the presence of either boat or ambient reef noise in a shallow (2 m) sand patch (Fig. S1). Fish were placed into the jars, given 10 min to habituate to the jar before being clipped onto the stage of the startle apparatus, where they were given a further 5 min habituation. The noise treatment (ambient, 2-stroke or 4-stroke boat noise) was started 1 min prior to the application of the startle stimulus. The startle apparatus consisted of a looming stimulus (wooden rod with a black tip) that travelled at 3.3 m/s (±0.3 SD) towards the jar, and which stopped 2 cm before contact (Fig. S2). This often elicited a C-start response^48^, but also caused other, less overt, responses such as backing away. The stimulus was triggered remotely and the fish’s response was recorded with a GoPro (3 silver) at 120 fps. Looming stimuli have typically been used to provoke a startle response from acclimated fishes (e.g.,^49^) and a similar setup was used in a recent study of boat sound^24^. The mean oxygen use measured with an oxygen electrode (OXI 340i from WTW, Germany) within the jars over the experimental period averaged 5.2% (±1.9 SD, n = 9), indicating that oxygen within the confined space did not reach a level that was likely to affect performance.

### Statistical analyses

A one-factor multivariate analysis of variance (MANOVA) was undertaken to determine whether there was a difference in fish behavior in the presence of motorboats prior to addition of any treatment odours (n = 59–60 fish). Data for 2- and 4-stroke motorboats were analysed separately as they were not collected at the same time. In the data for the 2-stroke experiment, two fish (out of 60) had very low boldness scores (0.2, 0.3 on a 0–3 index) that were statistical outliers (Grubb’s tests P < 0.0001) so were removed from the analysis. ANOVAs were conducted to determine the nature of any differences found by MANOVA, and if significant were followed by Tukey’s HSD post-hoc means comparisons.

To determine whether boat noise affected the way fish respond to olfactory risk cues, a MANOVA was undertaken using the fixed factors Acoustic treatment (ambient, 2-stroke and 4-stroke engine), and Odour (seawater, heterospecific skin extract, conspecific skin extract). The difference between the values of the behavioural variables before and after the introduction of treatment odours was used as the data for analyses (n = 27–33 fish). Proportional data were used for total distance moved to reduce the high variability between individuals in activity, which is typical for behavioural variables. Once again, data for 2- and 4-stroke motorboats were analysed separately as they were not collected at the same time, and ANOVAs were conducted to determine the nature of any differences found by MANOVA. If ANOVA found significant effects these were elucidated using Tukey’s HSD post-hoc means comparisons. Assumptions of the tests were examined using residual analyses.

The startle reactions were tested within each engine type, comparing the frequency of occurrence of a response between boat noise and ambient sound using 2 × 2 contingency tables. The variables analysed were (a) the number reacting to the looming stimulus, (b) of the ones that did react, the number that undertook a C-start fast-start response, (c) of those that undertook a C-start response, how many turned toward the looming stimulus compared to those that turned away (directionality) (n = 17–21 fish).

## Results

### Soundscapes

**Figure 2:**
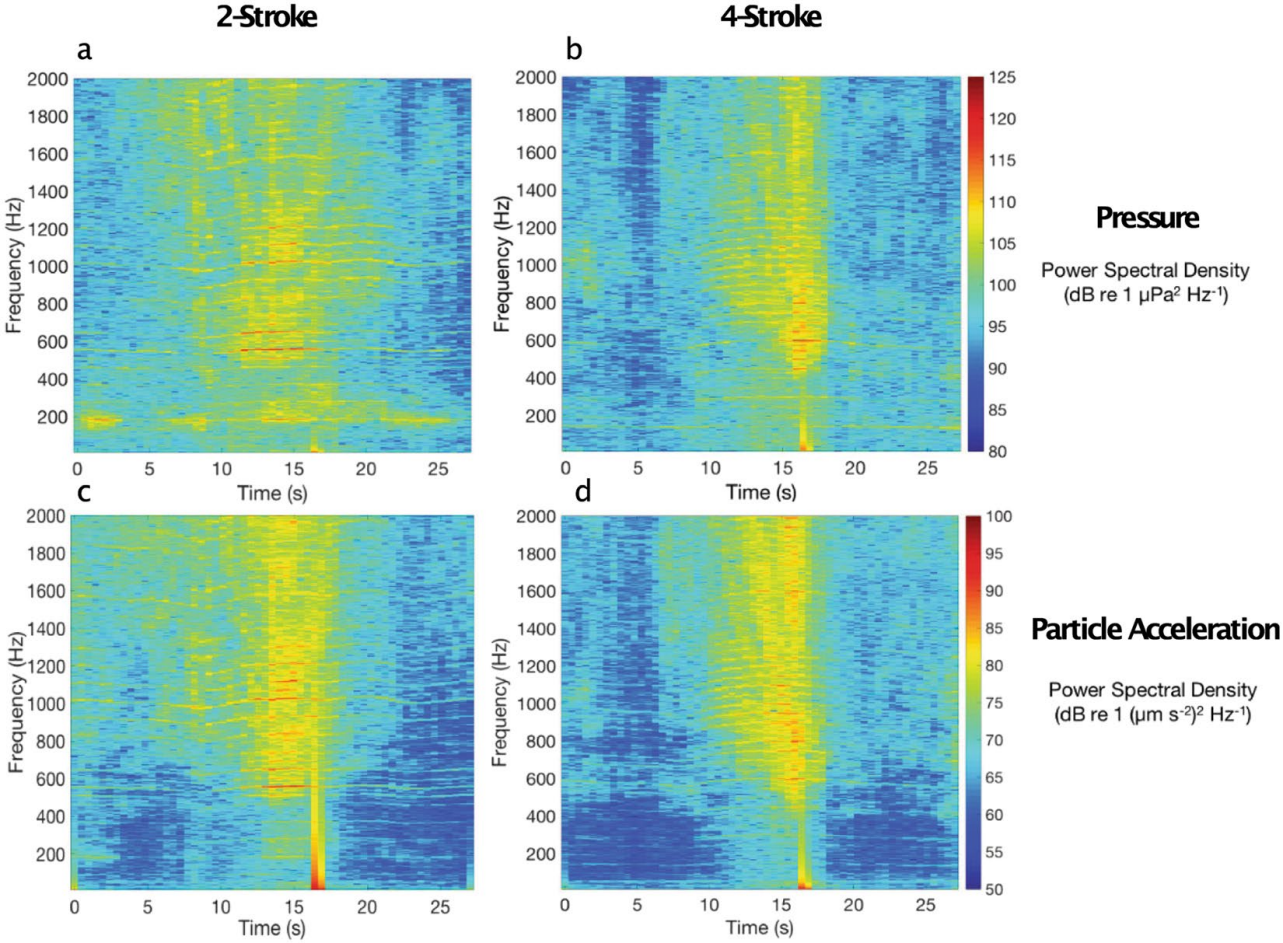
Representative spectrograms of individual motorboat passes for (**a**) 2-stroke and (**b**) 4-stroke engines in the pressure domain; and the same (**c**) 2-stroke and (**d**) 4-stroke passes recorded as monoaxial particle acceleration. Fast-Fourier Transformation = sampling rate (44100 Hz), Hamming window, 0–2000 Hz.

The acoustic pressure and particle acceleration conditions were markedly different between the ambient reef sound and the two boat noise treatments across all frequencies (Fig. 1a,b). Spectra for the 2- and 4-stroke boats were very similar, but in general the boats powered by 4-stroke outboards were of lower intensity across the majority of frequencies. While acoustic conditions within the holding tanks were generally noisier than ambient reef conditions, they remained markedly less noisy than open water experimental boat noise conditions (Fig. 1a,b). Acoustic spectra from representative vessel passes show that the noise from the 4-stroke powered boats was temporally more discrete in both domains than produced by 2-stroke powered boats (Fig. 2). Noise over 80 dB occurred within an ~8 s period for 4-stroke powered boats, while similar noise occurred over >15 s when a 2-stroke engine was used. In addition, noise in the pressure domain at ~200 Hz occurred over the whole 30 s sampling window for the 2-stroke powered boat pass (Fig. 2a).

### Effect of motorboat noise on risk assessment

**Figure 3:**
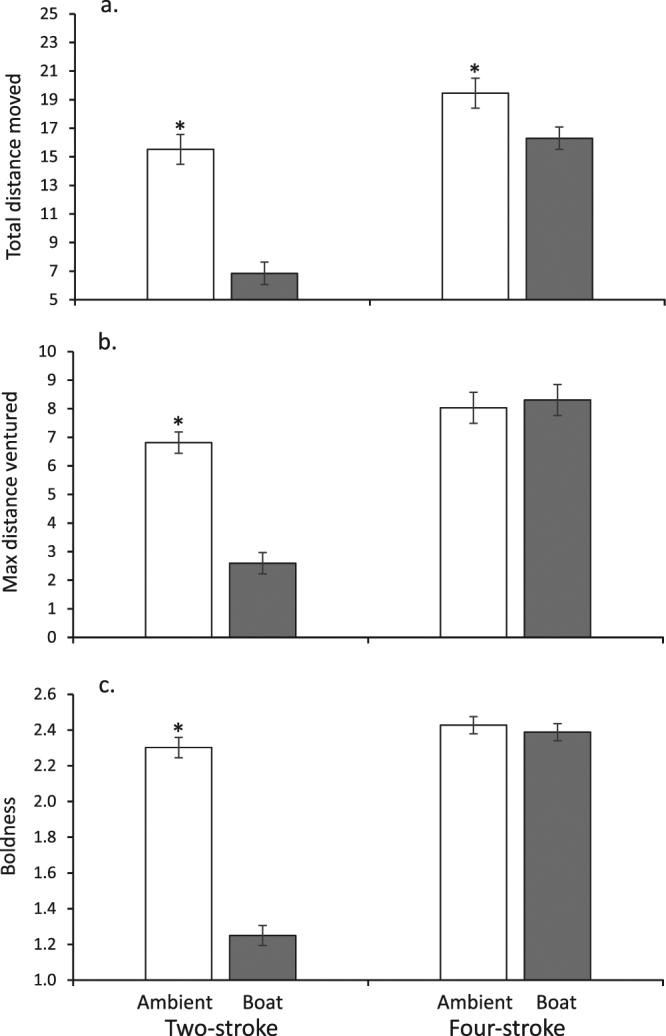
Comparison of the behavior of juvenile *Pomacentrus wardi* on isolated habitat patches that are exposed to ambient reef noise, or the noise of real motorboats powered by 2-stroke or 4-stroke 30 hp outboard engines. Three behaviours were assessed over a 3 min observation after a 10 min sound habituation period: (**a**) total distance moved (cm); (**b**) maximum distance ventured from the patch reef (cm); (**c**) boldness (an index between 0 and 3, see text for details). Asterisks indicate a significant difference between ambient and boat noise treatments. N = 59 to 60.

There was a significant effect of motorboat noise on the behavior of juvenile *P*. *wardi* prior to the introduction of any cues (MANOVAs: 2-stroke, F_3,116_ = 57.205, p < 0.0001; 4-stroke, F_3,114_ = 2.75, p = 0.046). ANOVAs indicated that there was a significant influence of 2-stroke boat noise on all three variables (total distance moved, F_1,118_ = 61.40, p < 0.0001; Max DV, F_1,118_ = 64.27, p < 0.0001; boldness, F_1,118_ = 173.09, p < 0.0001). Under noise from a 2-stroke, the behaviour was conservative, with values of total distance moved, Max DV and boldness being half of that of fish under ambient reef noise conditions (Fig. 3a–c). Under 4-stroke boat noise the difference in behavior between ambient sound and boat noise was due to a significant decrease in total distance moved when exposed to boat noise (F_1,118_ = 4.294, p = 0.040). Neither Max DV or boldness were significantly influenced by 4-stroke boat noise (p > 0.05; Fig. 3a–c).

**Figure 4:**
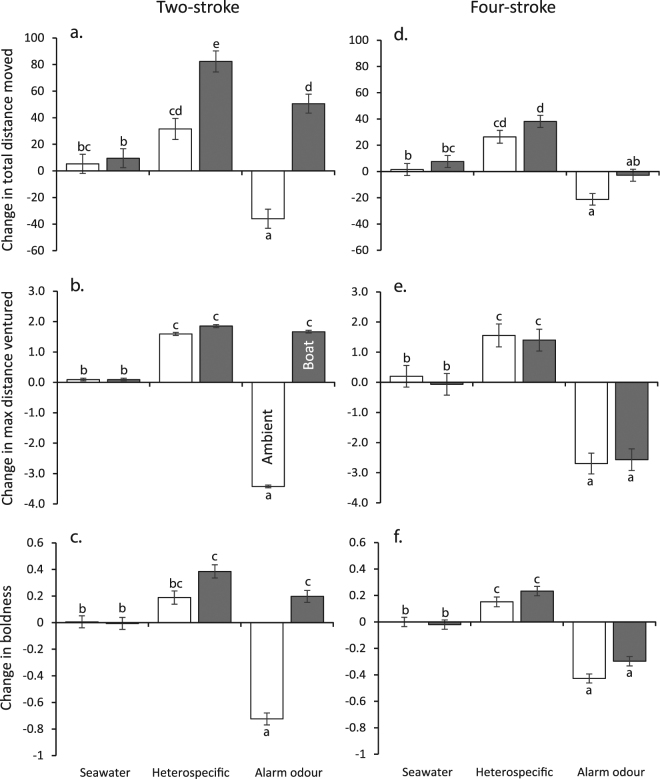
Change in behavior (mean ± SE) of juvenile *Pomacentrus wardi* on isolated patch reefs when exposed to one of either seawater, odour from damaged heterospecifics (Apogonids), or conspecific alarm odours, under ambient reef noise conditions (white bars) or when a motorboat powered by 2-stroke (**a**–**c**) or 4-stroke 30 hp outboard engine (**d**–**f**) was in the vicinity (grey bars). Behaviour recorded were: (**a**) total distance moved (proportion); (**b**) maximum distance ventured from the patch reef (cm); (**c**) boldness (an index between 0 and 3, see text for details). Letters on error bars are means groupings from Tukey’s HSD post-hoc tests. N = 27–33.

There was a difference in the way noise from the 2- or 4-stroke boats affected risk assessment in *P*. *wardi*. The odours to which fish were exposed influenced their behaviour, but the nature of the effect was dependent on whether fish were exposed to motorboat noise or ambient sound at the time of exposure to the olfactory cue (MANOVA, Acoustic treatment × Odour interaction, F_6, 346_ = 20.500, p < 0.0001). Total distance moved and boldness showed significant interactions between Acoustic treatment and Odour (Total distance, F_2,180_ = 21.23, p < 0.0001; MaxDV, F_2,180_ = 42.66, p < 0.0001; boldness, F_2,180_ = 58.85, p < 0.0001), and showed similar trends in how they were affected by the two factors (Fig. 4). Under a background of ambient sound, fish showed a slight increase in both behaviours when exposed to odours from damaged heterospecifics (though this was non-significant for change in boldness; Fig. 4). Fish showed a dramatic decrease in total distance travelled (36%), maximum distance ventured from shelter (3.5 cm) and boldness (30%) when exposed to odours from damaged conspecifics (Fig. 4). However, when under a background of 2-stroke boat noise, fish displayed an increase in total distance travelled, Max DV and boldness when exposed to damaged released odours, regardless of whether they originated from hetero- or conspecifics (Fig. 4a–c).

In contrast to the 2-stroke outboard, the way fish reacted to odours was not affected by whether they were exposed to ambient reef sound or noise from a motorboat powered by a 4-stroke outboard (MANOVA, Acoustic treatment x Odour interaction, F_6,346_ = 0.752, p = 0.608). In the trials with the 4-stroke engine, fish under ambient sound conditions showed a similar pattern of response to the different odours to that of ambient fish in the 2-stroke experiment. Fish showed an increase in distance moved, Max DV and boldness in response to heterospecific cues, while displaying a marked reduction in all variables in response to conspecific damage odours (ANOVA Odour treatment: total distance moved, F_2,174_ = 47.20, p < 0.0001; Max DV, F_2,174_ = 67.26, p < 0.0001; boldness, F_2,174_ = 125.96, p < 0.0001; Fig. 4a–c). There were significant differences for two out of three variables in response to background noise treatment. Fish showed marked increases in total distance moved and boldness after exposure to olfactory cues (compared to controls) when exposed to the 4-stroke outboard (Sound treatment: total distance, F_1,174_ = 10.344, p = 0.001; boldness, F_1,174_ = 4.879, p = 0.028), but this was not a trend repeated in Max DV (F_1,174_ = 0.109, p = 0.742).

### Startle response

**Figure 5:**
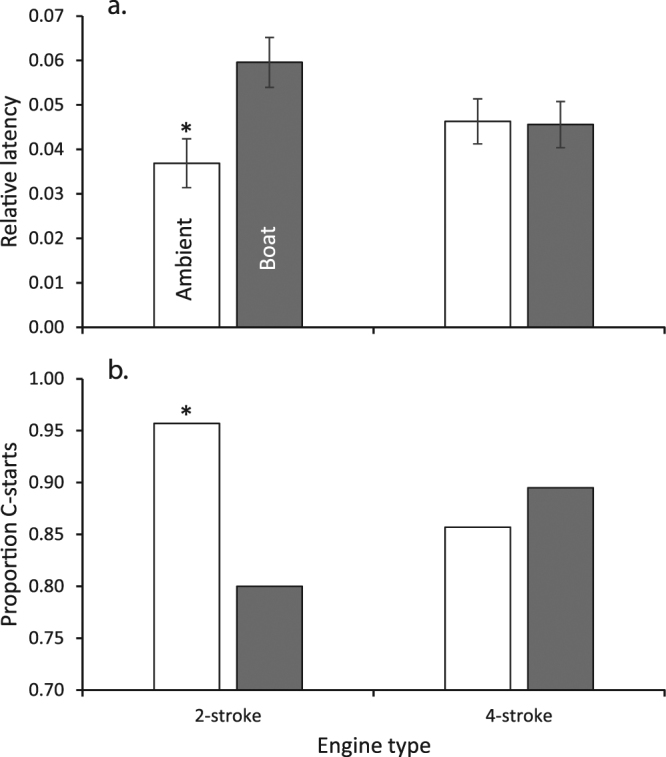
Startle response of *Pomacentrus wardi* when under conditions of ambient reef noise or noise from a motorboat powered by either a 2-stroke or 4-stroke outboard engine. Variable shown are: (**a**) mean latency to respond (±SE) to the looming stimulus relative to the fastest fish (s); (**b**) proportion of fish that undertook a C-start fast-start response when confronted with a looming stimulus (of the ~71% that reacted). Asterisks represent significant differences between ambient and boat treatments. N = 17–21.

The startle response was significantly affected by noise from 2-stroke boats when *P*. *wardi* were confronted with a looming stimulus. While there was no difference in the proportion that reacted to the stimulus, with a mean of 71% reacting (2-stroke, *X*^2^_1df_ = 0.037, p = 0.849; 4-stroke, *X*^2^_1df_ = 0.35, p = 0.554), there were differences in the way they responded. The time to react to the looming stimulus (latency) differed between treatments, with fish reacting almost 40% slower when exposed to a 2-stroke engine (F_1,39_ = 8.461, p = 0.006; Fig. 5a). In contrast, there was no difference in response latency when fish were exposed to ambient sound and noise from a boat powered by a 4-stroke engine (F_1,33_ = 0.010, p = 0.921; Fig. 5a).

The proportion of fish undertaking a C-start was lower during exposure to a 2-stroke engine, but not in the presence of a 4-stroke engine compared to ambient noise controls (Fig. 5b; 2-stroke, *X*^2^_1df_ = 4.823, p = 0.028; 4-stroke, *X*^2^_1df_ = 0.129, p = 0.720). Moreover, the proportion of fish that turned towards the looming stimulus was higher in the presence of 2-stroke engine noise compared ambient controls (20% vs 0%, *X*^2^_1df_ = 5.135, p = 0.023), but this was not the case when exposed to the noise from a 4-stroke engine (9.5% vs 4.7%, *X*^2^_1df_ = 0.359, p = 0.549).

## Discussion

Many studies have found that anthropogenic noise affects communication, movement patterns and foraging^2,9,50,51^, but it is often difficult to link these effects to individual fitness or population-level repercussions^24^. Studying antipredator behaviour offers a direct link to individual fitness since a reduced likelihood of escape directly affects survival^46^. Our study found that noise produced from small motorboats impacted the behaviour of juvenile damselfish by affecting the way they assess risk and their ability to detect and avoid a strike, and this should have a marked impact on survival. Interestingly, the noise produced by boats with 2-stroke engines dramatically affected all measured behavioural and performance variables, while quieter 4-stroke engines of a similar size (30 hp) had a detectable yet negligible effect on fish behaviour, or the fish’s detection and response to threats. These results underscore the potential for boat noise to alter the way fish use space and balance risk, which will likely impact on fitness.

The present study supports a growing body of research that suggests that small motorboats powered by 2-stroke engines have sub-lethal and lethal impacts on the physiology and behavior of marine invertebrates and fishes. Impacts include reduced acoustic acuity^19,25^, truncated embryonic development and survival^52^, increased oxygen consumption and reduced efficacy of startle responses leading to decreased survival^24^.

Few studies have examined how anthropogenic noise affects the antipredator responses of marine organisms, despite these behaviours being critical to survival. Four previous studies, which have used playback of anthropogenic (motorboat or ship) noise, have supported our field observations that noise disturbance can affect the reaction of animals to a looming stimulus (crabs^53,54^, eels^55^; sea bass^56^). Field and laboratory trials showed that the strike success of the dottyback, a voracious predator of juvenile fishes^57^, increased in the presence of boat noise disturbance^24^. Little is known of the response of different species to acoustic disturbance, but in the only study to compare the responses of two sympatric species to the playback of ship noise, Voellmy *et al*.^58^, found that the speed to undertake a startle response induced by a simulated bird attack was increased in the stickleback, but was not affected in the minnow in the presence of additional noise. These results suggest the outcome of predator–prey interactions will depend on the extent to which each species is affected by noise, and how noise affects the way individuals balance vigilance against other fitness-promoting behaviours, such as foraging.

The innate antipredator reaction to alarm odours is central to how many aquatic organisms, such as amphibians, fishes and some invertebrates, assess and learn risk^26,59,60^. While fish under the influence of 2-stroke motors were slightly more conservative in their space use and behaviour than reef noise controls, fish appeared to misinterpret the information from the damaged conspecifics and became more active, responding to the alarm odour with a feeding response. This misinterpretation takes place at a critical time when the occurrence of an alarm odour reliably indicates that there is a predator in the immediate vicinity that has just captured or attacked a conspecific, and so misjudging the information may have mortal consequences. An important mechanism of learning new risks is through the coincidence of the alarm odour with novel cues, whether they be visual, olfactory or mechanical (vibrations such as sound); and it is this coincidence that labels the novel cue as a threat^61^. The frequency of co-occurrence can then increase or decrease the importance of the threat, resulting in a graded and threat-sensitive response^26,62^. Further research is required to determine whether a loss of the antipredator response to alarm odours in the presence of 2-stroke engine noise may nullify its crucial role in this sophisticated threat-cataloguing mechanism.

Our findings show that when fish were exposed to 2-stroke boat noise alone the fish became less bold, in keeping with one previous study^63^. However, when noise and alarm odour were combined the fish became bolder. We believe this conflicting response is due to the boat noise confusing fish as to what the odours should represent. Interestingly, a very similar effect is found when *Pomacentrus amboinensis* recruits respond to alarm odours on degraded coral habitat patches, compared to their reaction on live coral patches^28^. We believe they are no longer able to judge risk effectively and the odour triggers a feeding response, which has previously been observed in the field in response to odours from damaged heterospecifics (e.g., apogonids^21^). We interpret this finding as the response of a disoriented fish to a misidentified cue and as such it represents a maladaptive response.

It is perhaps unsurprising that different sources of noise have different effects on animals^8,10^. However, this study is the first to demonstrate this for the outboard engines that power many recreational motorboats and small boats within the commercial line-fishing fleets that target coral reefs^31^. Our results demonstrate that 2- and 4-stroke engines, while having the same power (30 hp), have slightly different acoustic frequency power spectra and this is sufficient to lead to major differences in the way juvenile fish use space, respond to a chemical indicator of a threat and react to a startle stimulus. De Robertis and Handegard^64^ found differences in the way walleye pollock in the Bering Sea responded to noise from noise-reduced and standard vessels, but the effect was variable and context-dependent. Fish can react so strongly to low frequency sound^65^ that low frequency sounds have been used to induce fish avoidance behaviours in the vicinity of power plant water intakes^65,66^. Interestingly other species do not appear to be able to receive low frequency sound even within the same family^67^. Recently, Parmentier *et al*.^68^, exposed 20 species of 10 different families of settlement-stage coral reef fishes to playback of natural habitat soundscapes of different power-frequency spectra and found that there were species-specific differences in their response to the sounds when placed in a field choice chamber. The findings of the present study, and others generally, highlight the disturbance-specific (and possibly context-specific) nature of the response of animals to non-natural noise, and the necessity for further research to examine the context- and ontogenetic-specificity of these effects.

The pressure and power spectra of the outboard powered boats differed markedly from ambient reef sound, but did not differ dramatically from one another, despite strongly affecting the behaviour of the target fish. This finding suggests that the target fish were attuned to the subtleties in the difference between noise spectra. The spectra clearly show that the two-stroke powered boats were up to 10 dB greater in pressure and particle motion than the noise produced by the boats with 4-stroke engines. Moreover, the temporal power spectra indicates that the 2-stroke engines are “rattly”, probably because they have less cylinders firing with more power. In contrast, 4-strokes “hum”, with more cylinders firing with less power per piston stroke. Surprisingly, we are unaware of any research that previously compares the acoustic characteristics of different types of outboard motors. Clearly, more research is required to determine the components of noise that most affect the physiology and ecology of fishes.

Our study used a single noise exposure period (i.e., duty cycle) of ~9 min and found that this was sufficient to cause a change in risk assessment for the newly settled damselfish. Our exposure period is potentially equivalent to the magnitude of exposure expected in a narrow channel near a village or urban centre, or the entrance to a small marina. The noise was produced by the passage of real boats, rather than playback, or from an engine secured at set distance from the experimental fish (e.g.,^69^). While there is some evidence that this life-stage of fish may be able to habituate behaviourally to chronic boat noise^63^, it is likely that in real situations the acoustic disturbance will be unpredictable in intensity and timing, making habituation less likely. Moreover, given our fish received 1–3 min to habituate to the noise, this would have likely reduced any immediate fright response associated with an acute disturbance, meaning that our findings potentially represent an underestimate of the magnitude of response to acoustic disturbance from 2- and 4-stroke outboard powered boats.

Small motorboats by their very nature enable users to access the shallow waters of coastal zones and it is here where most of the boating activity is concentrated for transport, fishing and recreation. The human quest to visit, explore, map, and exploit areas of high biodiversity means that small motorboats are used where they affect the largest number of aquatic organisms. On coral reefs small motorboats often pass directly over or adjacent to coral reefs and their inhabitants. The mechanisms underlying the detrimental effects of 2-stroke engines on juvenile fish are currently poorly understood. In part it involves stress^24^, but the extent to which distraction, masking and damage of the acoustic system play a role are unknown for these vulnerable life stages and await further study. Our understanding will also benefit from an appreciation of the species-specific solutions to the alterations of the sensory inputs from vibrations and potential compensation by other senses and behavioural solutions. While this research was only conducted on one species and a specific life-stage, the finding that 4-stroke outboard motors have a negligible impact on the vulnerable juveniles compared to commonly used 2-stroke engines potentially provides marine resource managers with a powerful tool by which they can reduce the detrimental effects of noise from small motor craft on inshore fish communities.

### Ethics statement

All work carried herein was in accordance with the James Cook University Animal Ethics guidelines (JCU Animal Ethics approvals A2005 and A2080, collection permit G12/35117.1) and with University of Exeter Ethical Committee Approval (2013/247).

### Data availabilty

Data can be found at: 10.4225/28/5a8e04ef8103e.

## Electronic supplementary material

Site information and startle appartatus
