# Supplementary material for: Boat noise impacts risk assessment in a coral reef fish but effects depend on engine type
Source: Sci Rep. 2018 Mar 1;8:3847. doi: 10.1038/s41598-018-22104-3 (PMC5832755; doi:10.1038/s41598-018-22104-3)
Supplement: Supplementary file 1 — Site information and startle appartatus [file 41598_2018_22104_MOESM1_ESM.pdf]

## Supplementary file

**Boat noise impacts risk assessment in a coral reef fish but effects depend on engine type**

Mark I. McCormick, Bridie J. M. Allan, Harry Harding, Stephen D. Simpson

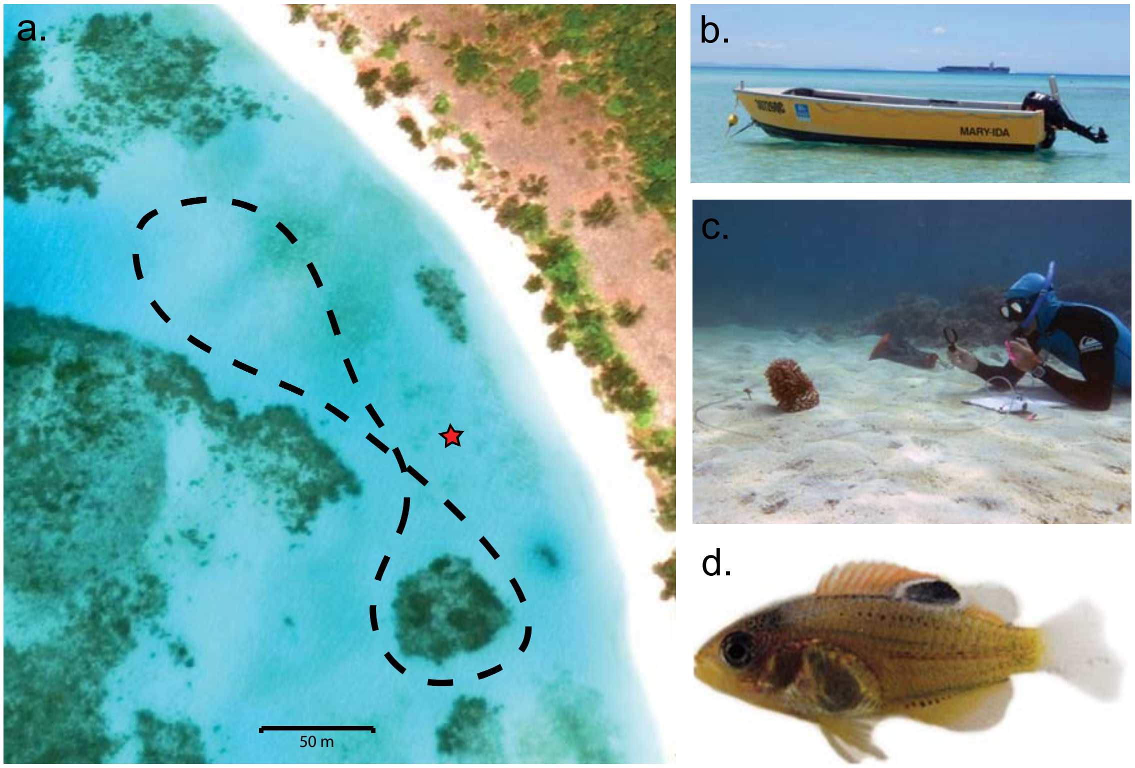


**Fig. S1.** a) Casuarina beach, a shallow sand area at the leeward side of Lizard Island on the northern Great Barrier Reef, and the location for the study. The track of the motorboats is shown (dotted line) and the position of the behavioural study site (star). b). An example of the 5m aluminium dinghies used for the study. c) Method used for behavioural assessment showing the odour release tube upcurrent of the coral patch and the observer using a magnifying glass to observe the fish (not at the actual study site). d) the juvenile study species, *Pomacentrus wardi*. Photographic credits: M. McCormick.

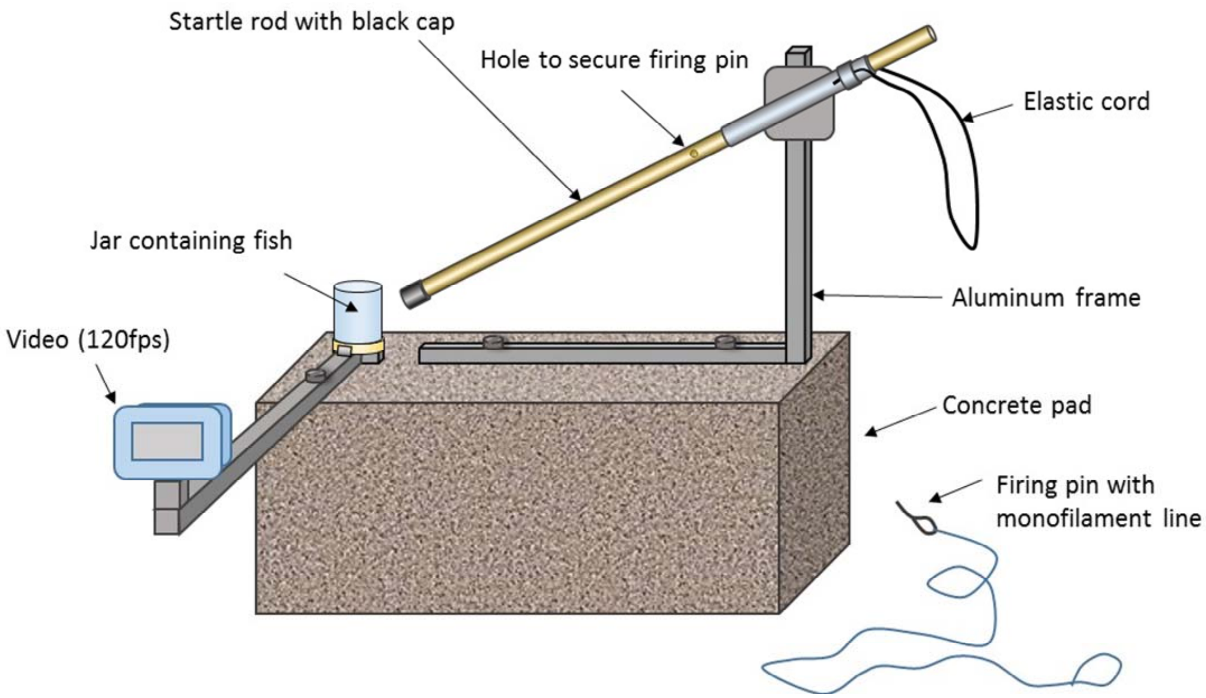


**Fig. S2.** Field fast-start apparatus used to assess the fast-start response of juvenile damselfish when exposed to ambient reef sound, or the noise from small boats powered by 2-stroke or 4-stroke outboard engines.
